# Soil nitrogen determines greenhouse gas emissions from northern peatlands under concurrent warming and vegetation shifting

**DOI:** 10.1038/s42003-019-0370-1

**Published:** 2019-04-18

**Authors:** Junwei Luan, Jianghua Wu, Shirong Liu, Nigel Roulet, Mei Wang

**Affiliations:** 1 International Centre for Bamboo and Rattan, 100102 Beijing, PR China; 2 Environment and Sustainability, School of Science and the Environment, Grenfell Campus, Memorial University of Newfoundland, Corner Brook, NL A2H 5G4 Canada; 3 The Research Institute of Forest Ecology, Environment and Protection, Chinese Academy of Forestry, 100091 Beijing, PR China; 4 Department of Geography and School of the Environment, McGill University, Montreal, QC H3A 2K6 Canada; 5 School of Geographical Science, South China Normal University, 510631 Guangzhou, PR China

**Keywords:** Ecosystem ecology, Climate-change ecology, Wetlands ecology, Biogeochemistry

## Abstract

Boreal peatlands store an enormous pool of soil carbon that is dependent upon – and vulnerable to changes in – climate, as well as plant community composition. However, how nutrient availability affects the effects of climate and vegetation change on ecosystem processes in these nutrient-poor ecosystems remains unclear. Here we show that although warming promoted higher CH_4_ emissions, the concurrent addition of N counteracted most (79%) of this effect. The regulation effects of the vegetation functional group, associated with the substrate quality, suggest that CH_4_ emissions from peatlands under future warming will be less than expected with predicted shrub expansion. In contrast, N_2_O flux will be enhanced under future warming with predicted shrub expansion. Our study suggests that changes in greenhouse gas emissions in response to future warming and shifts in plant community composition depend on N availability, which reveals the complex interactions that occur when N is not a limiting nutrient.

## Abstract (web summary)

Junwei Luan, Jianghua Wu et al. examine how nutrient availability affects biotic and abiotic variables in boreal peatlands. They show that changes in greenhouse gas emissions in response to plant community composition and warming depend on nitrogen availability.

## Introduction

Northern peatlands store ~30% (~600 Gt) of the world’s terrestrial soil carbon (C)^1^, equivalent to half of the total atmospheric C^2^. This enormous store of soil C results from persistently greater rates of plant production than decomposition, due to the high water content, poor nutrient^3^, and recalcitrant litter such as *Sphagnum* moss^4^, all of which reduce decomposition. However, the anoxic conditions of northern peatlands make them a global source of methane (CH_4_), annually releasing 10–25 CH_4_-C Tg (12.2% of the global total) into the atmosphere^5^.

Carbon dioxide (CO_2_), CH_4_, and nitrous oxide (N_2_O) are the three most important greenhouse gases (GHGs), after water vapor. Emission of GHGs by northern peatlands are tightly coupled to climate change through the impact of climate on peatland hydrology and plant community composition^6,7^. One concerning factor is that the majority of peatlands are located in northern high latitudes where the climate is experiencing a greater rate of change than in the past^2^, and climate warming is expected to increase nutrient mineralization from soil organic matter^8^. Moreover, the C pool in northern peatlands is susceptible to changes in climate^6^ via changes in temperature, soil water content, and soil nutrients. Further, climate warming can alter the vegetation composition, e.g., shrub expansion in tundra areas^9–11^, shifting the dominance of plants from *Sphagnum* to a graminoid-dominated system in poor fens^12^, or leading to the loss of selective plant species^9,11,13,14^. Land use change also affects the plant community composition. For example, burning and grazing promoted fast-growing graminoids over slower-growing ericaceous shrubs and mosses^15^, while drainage reduced the coverage of *Sphagnum* moss on hummocks that facilitated the invasion of sedges on lawns in a poor fen^16^. Previous studies have shown the rapid response of C^17,18^, even subsurface peat^19^ or methane emission^17^, to manipulative warming from peatlands. The change in vegetation composition or biodiversity loss can also exert severe impact on both short-term C fluxes^20^ and long-term soil C storage^21–23^. For example, the presence of graminoids (sedge-dominated in this case) has previously been demonstrated to positively impact CH_4_ flux, either by facilitated transportation due to the presence of aerenchymatous tissues^16,24,25^ or by increased supply of available substrates for methanogenic activities^26,27^. The complex interactive effects between abiotic and biotic variables on ecosystem C processes are becoming more apparent^28^. It was illustrated that the effect of warming on GHG fluxes in peatlands are modulated by plant community composition^25^.

Northern peatlands tend to be nutrient-limited with slow rates of decomposition^3^. Carbon storage in boreal ecosystems is thought to be constrained ultimately by C–nutrient interactions because plant production is usually nitrogen (N)-limited^29^. It has been found that northern peatlands with different soil N concentrations show very different responses to increases in temperature^30^. Different vegetation types, for example shrubs, sedges, and *Sphagnum* mosses, have been illustrated to show disparate responses to experimental N addition^31,32^, e.g., increased above-ground vascular plant biomass (e.g., *Vaccinium oxycoccus*^33^), reduced peat-forming *Sphagnum*^32^, or change in species composition^34^. Therefore, we hypothesized that the combined effects of warming and vegetation shifting on ecosystem processes largely depend on N availability. Unraveling the underlying mechanism is crucial because the global N deposition is predicted to double by 2050^35^.

Here, we report the results from a manipulative experiment based on a fully factorial design enabling us to examine the interactive effects of passive warming and plant community composition on GHG emissions under both N-ambient and N-added conditions from a boreal peatland (Supplementary Figure 1 for the experimental design). We expected the system would experience a maximum response in the short term as we were suddenly inducing a disequilibrium. Further, we acknowledged that the short-term responses would be transient until a new equilibrium is reached in the long run. However, we argue that the transient results would be still very useful because they would offer us the information of whether the system would arrive at the same equilibrium or move to a new equilibrium in the long run. Hexagonal open-top chambers^36^ were placed on half of the experiment plots in an area of oligotrophic blanket bog in Newfoundland, Canada, to achieve an ~1.2 ℃ increase in soil temperature at 5 cm depth during the mid-day period. Warmed or ambient temperature plots were manipulated by both the addition of N and the removal of selected vegetation types, including graminoids and shrubs, while the moss layer was kept intact to minimise the soil disturbance. The experiment was carried out for 2 years. We present results from field measurements of GHG fluxes, namely CO_2_ (here represented by ecosystem respiration), CH_4_, and N_2_O fluxes during the second growing season. Our results demonstrate that the increase in CH_4_ emissions from northern peatlands in response to climate warming may be substantially smaller than previously predicted with elevated N deposition; the projected shift to increased shrub cover in boreal peatlands may lead to a less pronounced response of CH_4_ emissions to climate warming, but a stronger N_2_O exchange between the atmosphere and peatland ecosystems under the projected warming along with increasing atmospheric N inputs.

## Results

### Open-top chambers enhance air temperature

Open-top chambers statistically significantly increased soil temperature, on average, by 1.2 °C (*F*_1,384_ = 18.2, *P* < 0.001) at 5 cm depth and 0.44 °C (*F*_1,384_ = 29.8, *P* < 0.001) at 20 cm depth (Supplementary Tables 1 and 2; Supplementary Figure 1). We found no evidence that either N (with addition of 6.4 g N m^−2^ year^−1^; *F*_1,384_ = 0.05, *P* = 0.83) or vegetation manipulation (graminoid present/absent: *F*_1,384_ = 1.06, *P* = 0.31; shrub present/absent: *F*_1,384_ = 0.07, *P* = 0.79) affected soil temperature. All treatments of our experiment had no significant effects on water table depth (Supplementary Tables 1 and 2).

**Fig. 1:**
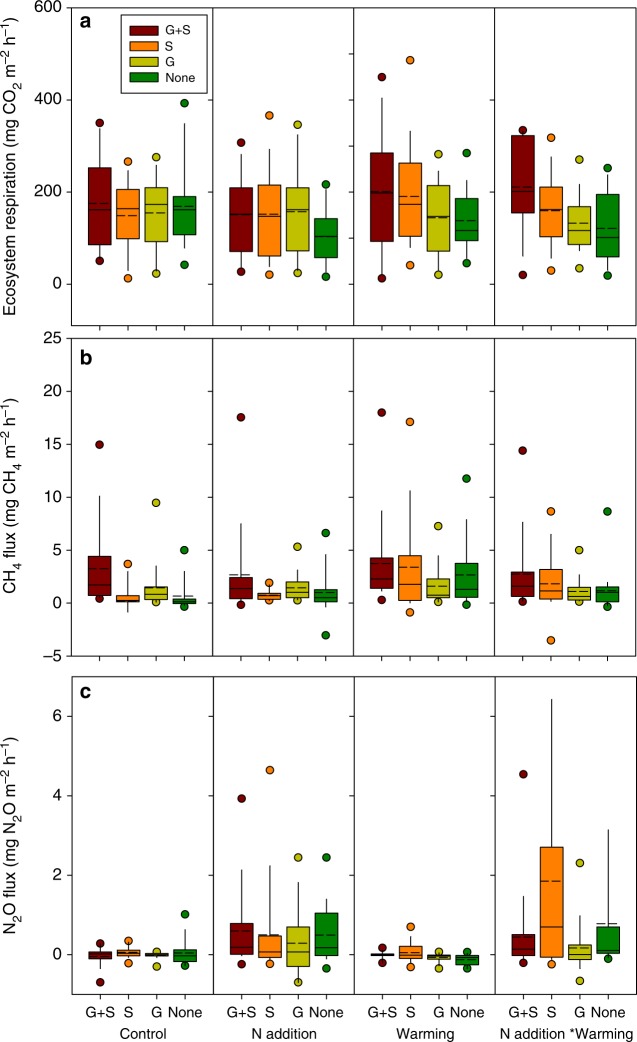
Ecosystem respiration (**a**), methane (**b**), and nitrous oxide (**c**) fluxes observed during the manipulation experiment year of 2015. Solid circles mean the ninety-fifth and fifth percentiles, the range of each column is from twenty-fifth to seventy-fifth percentile, the short dash in each column indicates the mean, the dash in each column is the median. G + S graminoids and shrubs both present, G graminoids only present, S shrubs only present, None no vascular vegetation present. Note: for all plots, the substrate layer of *Sphagnum* mosses remained intact

Nitrogen addition, warming, and vegetation removal had no significant effects on CO_2_ flux (Fig. 1a, Table 1). Although warming increased CO_2_ flux by 25%, from 162.1 ± 13.4 (SE) to 203.3 ± 14.1 mg m^−2^ h^−1^ for sites with both shrub and graminoid present (G + S), this increase is statistically not significant (*F*_1,83_ = 3.39, *P* = 0.06). A consistent seasonal pattern of CO_2_ flux among treatments was observed (Supplementary Figure 2), but there were no interactions between sampling time and treatments (Supplementary Table 3).

**Table 1:** Repeated measures ANOVA shows the effects of, and interactions between, N addition, warming and present/absence of plant functional groups, i.e., shrubs and graminoids, on ecosystem respiration, CH_4_ and N_2_O fluxes

Manipulated variables	Statistical parameters for dependent variables								
	Ecosystem respiration			CH_4_			N_2_O		
	*df*	*F*	*P*	*df*	*F*	*P*	*df*	*F*	*P*
	*n* = 333			*n* = 374			*n* = 304		
N added/no N added	1	0.16	0.70	1	1.51	0.23	1	**22.8**	**0.001**
Warmed/ambient temperature	1	0.01	0.93	1	**12.3**	**0.001**	1	0.17	0.69
Graminoid presence/absence	1	0.00	0.99	1	**5.50**	**0.02**	1	0.92	0.36
Shrub presence/absence	1	0.43	0.53	1	3.65	0.06	1	1.63	0.23
N added × warmed	1	0.35	0.57	1	**9.27**	**0.004**	1	**6.36**	**0.03**
N added × graminoids	1	0.09	0.76	1	1.22	0.28	1	1.99	0.19
N added × shrubs	1	0.08	0.79	1	0.05	0.82	1	0.45	0.52
Warmed × graminoids	1	0.10	0.76	1	**10.1**	**0.003**	1	**11.4**	**0.007**
Warmed × shrubs	1	0.15	0.71	1	3.57	0.06	1	**15.6**	**0.003**
Graminoids × shrubs	1	0.21	0.66	1	**4.71**	**0.04**	1	**5.93**	**0.035**
N added × warmed × graminoids	1	0.10	0.76	1	2.53	0.12	1	**5.39**	**0.043**
N added × warmed × shrubs	1	0.13	0.73	1	0.15	0.70	1	**5.75**	**0.037**
Warmed × graminoids × shrubs	1	1.92	0.20	1	3.83	0.058	1	0.76	0.403
N added × graminoids × shrubs	1	0.12	0.73	1	0.67	0.42	–	–	–
N added × warmed × graminoids × shrubs	1	0.83	0.39	1	0.55	0.56	–	–	–

Significant (*P* < 0.05) values are presented in bold

*df* degree of freedom, *F* F-value, *n* number of samples

### N addition counteracts warming-induced CH_4_ emission

Our analysis revealed significant treatment effects from both warming and plant community composition on CH_4_ emission (Fig. 1b, Table 1). Specifically, the warming doubled the overall mean CH_4_ emission from 1.39 ± 0.29 to 2.85 ± 0.39 mg m^−2^ h^−1^ regardless vegetation composition, and CH_4_ emissions were larger in the presence of graminoids (2.24 ± 0.23 mg m^−2^ h^−1^) than without graminoids (1.46 ± 0.21 mg m^−2^ h^−1^) independent of other treatments. Although N addition alone did not influence CH_4_ emission (*F*_1,374_ = 1.51, *P* = 0.23), we observed a significant combined effect of N addition and warming on CH_4_ emission (*F*_1,374_ = 9.27, *P* = 0.004). More specifically, N addition counteracted most (~79%) of the warming-induced increase in CH_4_ emissions (Fig. 1b) and led to an overall mean CH_4_ emissions rate of only 1.69 ± 0.27 mg m^−2^ h^−1^ (a 0.30 mg m^−2^ h^−1^ increase) for combined treatments (compared with 2.85 ± 0.39 mg m^−2^ h^−1^ for the plots receiving the treatment of warming independent of vegetation composition). On the other hand, we detected an interactive effect on CH_4_ emissions of warming with the removal of graminoid (*F*_1,374_ = 10.1, *P* = 0.003), with the lowest CH_4_ emission rates being measured in unwarmed plots without the graminoids (0.16 ± 0.47 mg m^−2^ h^−1^, a greater than 0.8 factor decrease) and the highest CH_4_ emission rates in warmed plots with intact vegetation (3.73 ± 0.87 mg m^−2^ h^−1^, Fig. 1b). Additionally, the presence of graminoids or shrubs interacted to affect CH_4_ flux (*F*_1,374_ = 4.71, *P* = 0.04). No interactions between N addition and vegetation manipulation were found to affect CH_4_ emissions (Table 1).

### Warming and shrub expansion enhances N_2_O flux

The N addition increased the N_2_O flux by 44-fold, from an overall mean of 0.011 ± 0.026 mg m^−2^ h^−1^ for the control treatment to 0.48 ± 0.11 mg m^−2^ h^−1^ for the N addition treatment (*F*_1,304_ = 22.8, *P* = 0.001; Table 1 and Fig. 1c). We did not detect a significant effect of warming alone on N_2_O flux (*F*_1,304_ = 0.17, *P* = 0.69). An interactive effect was observed between N addition and warming (*F*_1,304_ = 6.36, *P* = 0.03), wherein warming further enhanced the positive effect of N addition on N_2_O flux by 67% (Fig. 1c). The interactive effect between N addition and warming varied according to plant functional groups (Table 1), with the largest N_2_O flux being found at sites with the treatments of N addition and warming where only shrubs were present (1.85 mg N_2_O m^−2^ h^−1^) and the smallest at sites with the treatments of N addition and warming where only graminoids were present (0.17 mg N_2_O m^−2^ h^−1^, Fig. 1c). Significant interactions between warming and graminoids (*F*_1,304_ = 11.4, *P* = 0.007) and between warming and shrubs (*F*_1,304_ = 15.6, *P* = 0.003) were detected, suggesting the effect of warming on N_2_O flux was regulated by the removal of different plant functional groups (Table 1, Fig. 1c). The presence of graminoids or shrubs, without N addition and warming, also interacted to affect N_2_O flux (*F*_1,304_ = 5.93, *P* = 0.035).

### Vegetation regulates substrate quality altered by warming

**Fig. 2:**
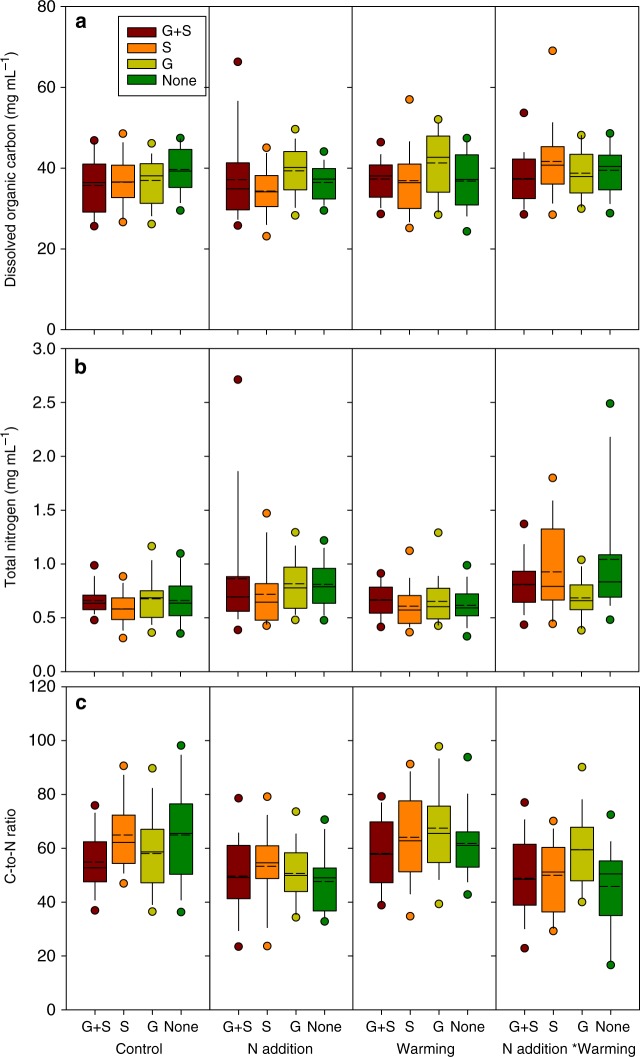
Dissolved organic carbon (DOC) (**a**), total nitrogen (TN) (**b**), and C/N ratio (**c**), for soil pore water under different treatments. Solid circles mean the ninety-fifth and fifthpercentiles, the range of each column is from twenty-fifth to seventy-fifth percentile, the short dash in each column indicates the mean, the dash in each column is the median. G + S graminoids and shrubs both present, G graminoids only present, S shrubs only present, None no vascular vegetation present

**Fig. 3:**
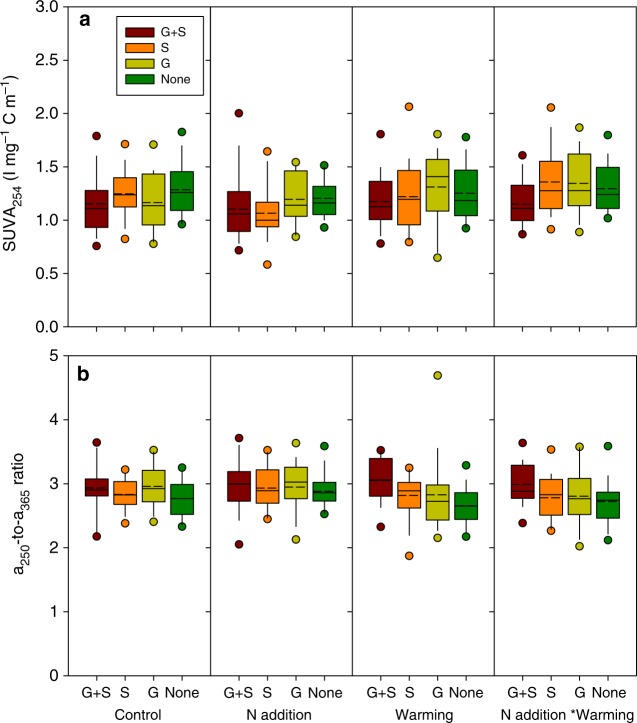
Dissolved organic carbon (DOC) composition indices SUVA_254_ (**a**) and a_250_/a_365_ (**b**) for soil pore water under different treatments. Solid circles mean the ninety-fifth and fifth percentiles, the range of each column is from twenty-fifth to seventy-fifth percentile, the short dash in each column indicates the mean, the dash in each column is the median. G + S graminoids and shrubs both present, G graminoids only present, S shrubs only present, None no vascular vegetation present

Warming significantly increased pore water dissolved organic C (DOC) from 35.7 ± 1.40 to 37.3 ± 1.01 mg L^−1^ (*P* = 0.04) (Table 2 and Fig. 2). N addition did not significantly change DOC (Table 2). A significantly higher DOC was observed in plots where shrubs were absent than in those where they were present (*F*_1,382_ = 3.48, *P* = 0.07, Table 2). Significant interactions were observed among warming, N addition, and graminoid removal on DOC (Table 2). Warming slightly increased DOC aromaticity, where peatland derived DOC with high aromaticity is often linked to low bioavailability^37^, indicated by specific UV absorbance at 254 nm, SUVA_254_ (*P* = 0.07), and graminoid removal significantly increased DOC weight-averaged molecular weight (indicated by a UV absorbance ratio between 250 and 365 nm, a_250_/a_365_, *F*_1,317_ = 6.69, *P* = 0.01, Table 2, Fig. 3). Our data indicated that N addition and warming interactively affected DOC, TN, C/N ratio, and the chemical composition of DOC with the effects being regulated by vegetation composition.

**Table 2:** Statistical analysis of the effects of, and interactions between N addition, warming, and present/absence of plant functional groups, i.e., shrubs and graminoids, on soil pore water dissolved organic carbon (DOC), total nitrogen (TN), C/N ratio, and SUVA_254_ and a_254_/a_365_ of soil pore water DOC

Manipulated variables	Statistical parameters for dependent variables										
	DOC			TN		C/N ratio		SUVA_254_		a_250_/a_365_	
	*df*	*F*	*P*	*F*	*P*	*F*	*P*	*F*	*P*	*F*	*P*
	*n* = 382			*n* = 382		*n* = 380		*n* = 317		*n* = 317	
N added/no N added	1	0.08	0.78	**17.6**	**<** **0.001**	**20.7**	**<** **0.001**	0.05	0.83	0.18	0.67
Warmed/ambient temperature	1	**4.50**	**0.04**	0.31	0.58	0.14	0.71	3.23	0.07	1.76	0.19
Graminoid presence/absence	1	0.26	0.61	0.13	0.73	0.21	0.65	0.84	0.36	**6.69**	**0.01**
Shrub presence/absence	1	3.48	0.07	0.10	0.76	0.28	0.60	2.81	0.10	3.05	0.09
N added × warmed	1	0.39	0.54	0.54	0.47	0.34	0.56	1.54	0.22	0.31	0.58
N added × graminoids	1	0.17	0.68	1.75	0.19	1.21	0.28	0.13	0.72	0.42	0.52
N added × shrubs	1	1.04	0.31	0.01	0.91	0.34	0.57	0.08	0.78	0.04	0.85
Warmed × graminoids	1	0.00	1.00	1.77	0.19	1.69	0.20	0.002	0.96	0.75	0.39
Warmed × shrubs	1	0.24	0.63	0.43	0.52	1.84	0.18	0.05	0.83	1.85	0.18
Graminoids × shrubs	1	1.32	0.26	2.08	0.16	**5.65**	**0.02**	0.51	0.48	0.06	0.81
N added × warmed × graminoids	1	**8.48**	**0.006**	1.79	0.19	0.18	0.67	2.19	0.15	0.05	0.82
N added × warmed × shrubs	1	0.74	0.40	0.02	0.88	0.73	0.40	0.16	0.70	0.14	0.71
Warmed × graminoids × shrubs	1	2.40	0.13	0.02	0.88	0.56	0.46	2.41	0.13	0.60	0.44
N added × graminoids × shrubs	1	0.00	0.10	1.02	0.32	0.68	0.41	0.12	0.73	0.08	0.78
N added × warmed × graminoids × shrubs	1	0.47	0.50	0.12	0.73	0.04	0.85	0.46	0.50	0.02	0.89

Bold values indicate the effect is statistically significant

## Discussion

**Fig. 4:**
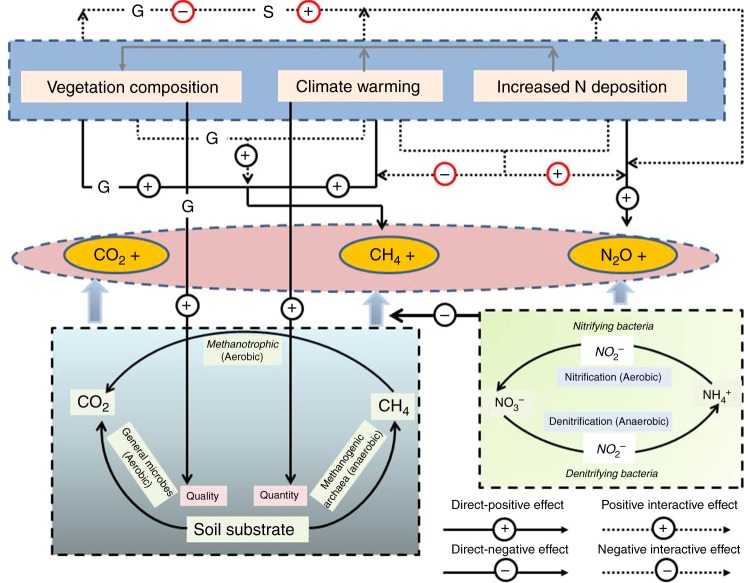
Schematic showing CO_2_, CH_4_, and N_2_O fluxes in northern peatlands and the potential independent and interactive impacts of warming, N deposition and plant community composition. The letter G or S on a line indicates the effect of the presence or absence of graminoids (G) or shrubs (S), respectively. The circles in red color indicate that the interactive effects revealed by this study but were unknown before

In our study, we manipulated both abiotic (temperature, N level) and biotic (presence or absence of vascular plant functional groups, i.e., shrubs and graminoids) factors to investigate the complex interactions regulating ecosystem processes, i.e., ecosystem respiration (*R*_eco_), CH_4_, and N_2_O fluxes. Our findings agree with our hypothesis that soil N condition regulates the independent, or the combined effects of warming and vegetation shifting on ecosystem processes. Based on our findings, we generated a conceptual model of GHG emission by northern peatlands (Fig. 4), which presents the complex regulating effects of soil temperature, soil moisture or water table depth, N conditions, and vegetation composition on GHG emissions. Particularly, this model emphasizes the interactive effects between warming and N deposition on CH_4_ and N_2_O fluxes, and between vegetation composition and N deposition on N_2_O flux, which were revealed for the first time through this comprehensively manipulative experiment that considered changes in climate, N availability, and vegetation at the same time.

The insignificant change of *R*_eco_ could be attributed to the slow stabilization of plant community composition to vegetation removal because we observed that warming slightly increased *R*_eco_ at the plots without vegetation removal, but this increase was not statistically significant (*P* = 0.06). Since our experiments have only run for 2 years, our findings must be viewed with caution. We still need to see if this insignificant change in *R*_eco_ would persist after a long-term period of warming and vegetation shifting (i.e., after 5 years’ treatment).

In contrast, the two potent greenhouse gases, CH_4_ and N_2_O, showed strong responses to the manipulations. Considering first CH_4_, warming unsurprisingly overall increased CH_4_ emissions by 105% regardless of vegetation treatment, which was consistent with previous research on peatlands^25,38^. The increased CH_4_ emissions resulted from warming may be due to increased methanogen activity, and substrate quantity (Table 2 and Figs. 2, 3) as a consequence of stimulated vegetation growth^39^. Previous studies have been contradictory regarding the impact of N addition on CH_4_ emissions, which have been shown as either a significant increase^40^ or a statistically insignificant change^41,42^. This has led to conflicting reports on the net impacts of N fertilizers on CH_4_ emissions^43,44^. Therefore, the insignificant effect of N addition on CH_4_ flux (Table 1) was not unexpected. However, N addition strikingly counteracted most (79%) of the warming-induced CH_4_ emissions (Fig. 1b), implying that future warming may not trigger as large a release of CH_4_ emissions from peatlands as has been anticipated^38,45^ in the presence of increasing N deposition from the atmosphere. Note must be made that the counteracting effect in our experiments could largely depend on the non-limited N supply in our plots as we used 10 times higher N than reported N deposition values in the N addition plots.

The mechanisms underlying the above counteracting effects may be two-fold. Given that the balance of methane production and consumption determines the net flux of CH_4_^46^, the suppression of methane production by nitrate^47,48^ could be enhanced by warming. Further, the methane oxidation by nitrogenous fertilizers^43,49^ may be stimulated under warmer conditions, which is less likely because CH_4_ oxidation rates in soils are theoretically less temperature dependent than CH_4_ production^50^. We also speculate that an increased supply of nitrite (NO_2_^−^) produced by nitrification or denitrification processes combined with a warmer climate would accelerate the process of anaerobic methane oxidation driven by oxygenic bacteria^51^. No matter which mechanism (if any) underlies the counteractive effect (Fig. 4), the negligence or unawareness on this issue^52^ leads to an over-estimation of future CH_4_ emission in response to warming based on our current understanding^53^. Caution has to be made that this is under the consumption of an unchanged water table since the influence of temperature on CH_4_ emissions is hydrologically dependent^54^. The regulating mechanism urgently needs to be disentangled through investigation of soil microbial dynamics under the conditions of warming and N addition.

The presence of graminoids (sedge-dominated in this case) has previously been demonstrated to positively impact CH_4_ emission, either by facilitated transportation due to the presence of aerenchymatous tissues^16,24,25^ or by increased supply of available substrates^26,27^. Consistently, graminoid removal that increased high molecular weight DOC and aromaticity (Table 2 and Figs. 2, 3) in our study may inhibit CH_4_ production. Here we observed that warming enhanced the facilitation effect of graminoids, suggesting that the direction of shifting in plant community composition induced by climate change^14^ determines CH_4_ emission. For example, the predicted shrub expansion in peatlands due to warming^11^ may interactively retard the warming-induced CH_4_ emissions. The regulation of substrate quality by a plant functional group may contribute to the interactive effect of warming and vegetation removal on CH_4_ emissions, since the sensitivity of organic matter decomposition to warming varies with substrate quality^55^.

N_2_O flux in our study was relatively low (0.011 ± 0.026 mg m^−2^ h^−1^), as would be expected in this nutrient-poor ecosystem, within the range (0.0004–0.034 mg N_2_O m^−2^ h^−1^) of previous reports from boreal peatlands^56^. Interestingly, warming alone did not affect the N_2_O flux, while interactively enhanced the effects of either N addition or functional group removal on N_2_O flux. In contrast with CH_4_, boreal peatlands would act as a stronger N_2_O source under future warming conditions with predicted shrub expansion^10,11^, while the N_2_O sink of sedge-dominated peatlands may be enhanced under future warming. These interactions (Fig. 4) have rarely been examined by field experiments or process-based modeling, which could lead to an over or underestimate of N cycling for northern peatlands that are being or will be dominated by specific plant functional groups under the condition of elevated N deposition. Although seasonal variations were observed for GHG fluxes (Supplementary Figure 2), no interactive effects of sampling time with treatments (Supplementary Table 3) were found in our data, which suggests that our treatment effects on GHG fluxes are independent of the measurement time.

In summary, our findings provide direct evidence that interactions occur among warming, N addition, and plant community composition to modify the GHG emissions from boreal peatlands. To the best of our knowledge, this is the first time that a counteractive effect of N addition on warming-induced CH_4_ emissions has been observed. If such a damping effect of N-related suppression of methane production was to occur globally, the increase in CH_4_ emissions from northern peatlands in response to climate warming may be significantly smaller than previously predicted, and thus may not cause as much positive climate feedback as anticipated. However, the universality and the experimental results need to be verified in different locations before the findings can be extrapolated to other peatlands. Moreover, the projected shift to increased shrub cover leads to a less pronounced response of CH_4_ emissions to climate change than expected by the decrease in substrate quality. In contrast, the projected shift to shrub cover may lead to stronger positive responses in terms of N_2_O exchange between the atmosphere and peatland ecosystems under predicted warming along with increasing atmospheric N inputs. We emphasize an urgent need to unravel the underlying mechanisms before incorporating the interactions between biotic and abiotic drivers into future modeling work. Our findings, for the first time, show a decisive control of soil N condition on the independent and interactive effects of warming and plant community composition on ecosystem processes. Moreover, our experiment can serve as a possible direction to ecosystem management in terms of mitigating climate change.

## Methods

### Study site

Our research site is located in an area of oligogenic, ombrotrophic blanket bog, in Robinsons, western Newfoundland, Canada (48°15′46′N, 58°39′ 21′W). The climate is oceanic temperate, with an annual rainfall of 1340 mm and annual average temperature of 5 °C (1981–2010)^57^. The mean pH (1:5 soil/water) at the site was 4.5 ± 0.01, and the mean peat depth of 3 m was derived from three random peat depth measurements at the site before the experiment was established. The site represents the typical type of peatland found on the island of Newfoundland, where the vegetation consists of an approximately equal biomass of graminoids (*Trichophorum cespitosum*, *Carex chordorrhiza*) and dwarf shrubs (*Gaylussacia baccata*, *Rhododendron groenlandicum*, *Andromeda glaucophylla, Ledum palustre* ssp.), with *Sphagnum* mosses (*Sphagnum* spp., *Hylocomium splendens*, *Aulacomnium turgidum*) providing the main matrix^39,57^.

### Experimental design

A factorial design comprising manipulation of temperature (warmed vs. ambient temperature), nitrogen (N; N addition vs. no N addition), and plant community composition (removal of graminoids only vs. removal of shrubs only vs. removal of both graminoids and shrubs vs. no vegetation removed) was established in the spring of 2014. The layout was 2 (Warming and ambient temperature) × 2 (N addition and no N addition) × 2 (shrubs present or absent) × 2 (graminoids present or absent) = 16 treatments. The experimental site comprised four blocks with 6 m apart from each other, and each block contained one each of all 16 treatments, randomly distributed (*n* = 64) (See Supplementary Figure 1 for the layout of the experiment design) 2 m × 2 m plot, with a buffer zone of intact vegetation of at least 2 m between adjoining plots.

Warming was achieved passively using hexagonal open-top chambers based on the ITEX design^58^. Each transparent section making up the hexagonal open-top chambers measured 80 cm along the bottom edge, 62.5 cm along the top edge, and 40 cm in height, to give an internal diameter of 1 m^2^. The chambers were constructed from 3-mm thick clear acrylic sheeting (Ridout Plastics, San Diego, U.S.), which allows 92% light transmission. The open-top chambers method offers a robust means to examine the effects of warming in remote environments without the need for a power supply^59^, and has been used frequently in arctic and peatland ecosystems^19,25,60^. The open-top chambers were fixed in place in early May of 2014 (in the center of each 2 m × 2 m plot), and the measurements ended in November 2015.

Air temperatures at vegetation canopy height were recorded continually at a 30-min time step using temperature loggers (Lascar Electronics, Salisbury, UK) and soil temperature at 5 cm and 20 cm depth was continuously recorded at a 30-min time step by soil temperature sensors (LI7900–180, LI-COR Inc., Lincoln, Nebraska, U.S.) connected to a Campbell data logger (CR1000, Campbell Scientific, Utah, USA) at two randomly chosen plots (one for the plot with warming and one for the plot with ambient temperature), and was also measured manually with a temperature probe in each plot during every gas sampling campaign. Water table levels were measured from dip-wells made of 1-m long perforated PVC pipe installed in each of the 64 experimental plots during every gas sampling campaign.

Annual background inorganic wet N deposition in the region is 0.5–0.6 g N m^−2^^61^. For the N addition treatment, from the start of the study we annually applied 6.4 g N m^−2^_,_ ~10 times the ambient annual wet N deposit. The rationale behind this quantity was to establish N-non-limited conditions for this nutrient-poor ecosystem, which is equivalent to the amount of high N addition level treatments used in another study in a northern peatland^32^. Nutrients were applied in soluble form as NH_4_NO_3_ in 2 L of water taken from an open pool close to the 2 × 2 m plots bimonthly from May to September each year. The same volume of open pool water was applied to the control sites.

Plant functional group manipulations were made only for the two dominant vascular vegetation types present at our sites: dwarf shrubs and graminoids. To avoid any soil disturbance, we did not manipulate the bryophyte/lichen functional type because of its substrate nature in this ecosystem. Vegetation removal was undertaken by hand from an area of 2 × 2 m. The shoots of shrubs and graminoids were cut back to litter layer level in early May 2014. Plots were left to settle for a year before sampling to minimize the effects of decomposition from roots. We did not include the first year’s data in our data analysis, since high variabilities of GHGs were observed for the first year (Supplementary Figure 3), and no significant treatment effect was statistically detected. Maintenance, such as removal of new shoots of removed vegetation types, was conducted regularly during each sampling campaign.

### Measurements of gas exchange

In each sampling plot, a PVC (polyvinyl chloride) collar with an inner diameter of 26 cm was permanently inserted into the peat to a depth of 10 cm in the spring of 2014. The upper part of the collar features a groove to accommodate the water seal needed for the chamber measurements. Care was taken during insertion to minimize disturbance and to avoid severance of large plant roots. Boardwalks were installed to prevent any damage to the vegetation, disturbance to peat gas storage, or emissions during site visits. We had in total six sampling campaigns at ~3-week intervals from June to October 2015. During each sampling campaign, measurements were done for all the plots and conducted between 10:00–15:00 local time during 2–4 days to avoid rainfall.

For ecosystem respiration (*R*_eco_), CH_4_, and N_2_O fluxes, gas samples were collected using opaque chambers 50 cm in height and 26.3 cm in diameter, fitted to the groove of the PVC collar, covered with aluminum foil to reduce any solar heating effect, and equipped with a capillary tube to maintain atmospheric pressure inside the chamber when sampling. Samples were taken immediately upon closure of the chambers and at 10 min., 20 min. and 30 min. after closure. Flux was calculated by linear regression using all four measurements sampled during the 30 min^39,57^. Because the light was blocked by the opaque chamber, no photosynthesis occurred. Thus, the flux calculated based on the change of CO_2_ concentration inside the opaque chamber can be considered to be the ecosystem respiration. Gas samples (25 mL) were taken from the chamber headspace using a gas syringe and injected into pre-evacuated 12 mL Exetainer vials (Labco, Lampeter, UK) for storage prior to analysis. Concentrations of CO_2_, CH_4_, and N_2_O were analyzed by gas chromatography using a Scion 456-GC (gas chromatograph; Bruker, Milton, Canada) equipped with a thermal conductivity detector for CO_2_, a flame ionization detector for CH_4,_ and an electron capture detector for N_2_O. For each sample, 5 mL of gas was injected into the chromatograph using an Autosampler (Combi PAL, Milton, Canada). The gas concentration was calculated using a calibration curve based on two certified standard gases, comprising 378 ppm and 0.303% CO_2_ (i.e., 3030 ppm), 2.52 and 17.7 ppm CH_4_, and 0.770 and 7.63 ppm N_2_O (Air Liquide, Canada). All fluxes were adjusted for field sampling temperature, headspace volume and chamber area^62^.

### Soil pore water chemical composition

Soil pore water samples were collected using a MacroRhizons sampler (Rhizosphere, The Netherlands) installed at ~10 cm depth in each plot. The sampler has a porous tip with an outer diameter of 4.5 mm and a pore size of 0.15 µm. MacroRhizons have a female luer lock fitting, which is suitable for creating a vacuum with a male luer lock syringe^63^. Samples were collected during each gas sampling campaign. DOC and dissolved total nitrogen (TN) analysis was conducted on a Shimadzu TOC-LCPH/TN analyzer (Shimadzu, Japan). Three injections of each sample were applied to calculate the average DOC and TN concentration for each sample.

The composition of DOC was assessed using three indices: specific UV absorbance (SUVA_254_), defined as UV absorbance at 254 nm normalized for the DOC concentration (1 mg^−1^ C m^−1^), which increases linearly with measured DOC aromaticity ^64^. Reported values of SUVA_254_ in natural waters usually range from 0.5 to 6 l mg^−1^ C m^−1^, equivalent to a range of percent aromaticity between 5 and 45%;^65^ UV absorbance ratio, between 250 and 365 nm (a_250_/a_365_), which is inversely related to the DOC weight-averaged molecular weight^66^ with reported values in the range three to eight; reported C/N ratio, assumed equal to the measured DOC/TN ratio.

### Statistical analysis

The effects of experimental warming, N addition, vegetation manipulation, and their interactions were analyzed by repeated measures ANOVA using IBM SPSS Statistics 20, with sampling date nested within sampling block as random effects. Vegetation manipulation effects were determined according to whether or not each of the two plant functional groups (shrubs or graminoids) was present. Data were checked for normality using the residual plots method and log-transformed where necessary before analysis.

### Reporting summary

Further information on experimental design is available in the Nature Research Reporting Summary linked to this article.

## Supplementary information

Supplementary Information

Description of Additional Supplementary Files

Reporting Summary

Supplementary Data 1

## Data Availability

The data that support the findings of this study are available from the corresponding author upon reasonable request.
