# Supplementary material for: Soil nitrogen determines greenhouse gas emissions from northern peatlands under concurrent warming and vegetation shifting
Source: Commun Biol. 2019 Apr 18;2:132. doi: 10.1038/s42003-019-0370-1 (PMC6472372; doi:10.1038/s42003-019-0370-1)
Supplement: Supplementary file 1 — Supplementary Information [file 42003_2019_370_MOESM1_ESM.pdf]

Supplementary materials:

Supplementary Figure 1 Appearance of an experimental block. Notes: Plot size: 2 m × 2 m, buffer zone between plots > 2 m. White, control (ambient temperature and no N addition); Orange, warmed by OTCs; Blue, N added; Green, warmed × N added. G+S, graminoids and shrubs both present; G, graminoids only present; S, shrubs only present; None, no vascular vegetation present. Left upper corner showed the air temperature between the ambient and inside OTC on June 20th and August 20th in 2014, as an example, based on half-hour step record, to show the passive warming effect of OTC in a daily time scale. We can clearly see that the air temperature inside the OTC (red dash) is higher than the control plot (black line), especially during daytime when our measurement was conducted.

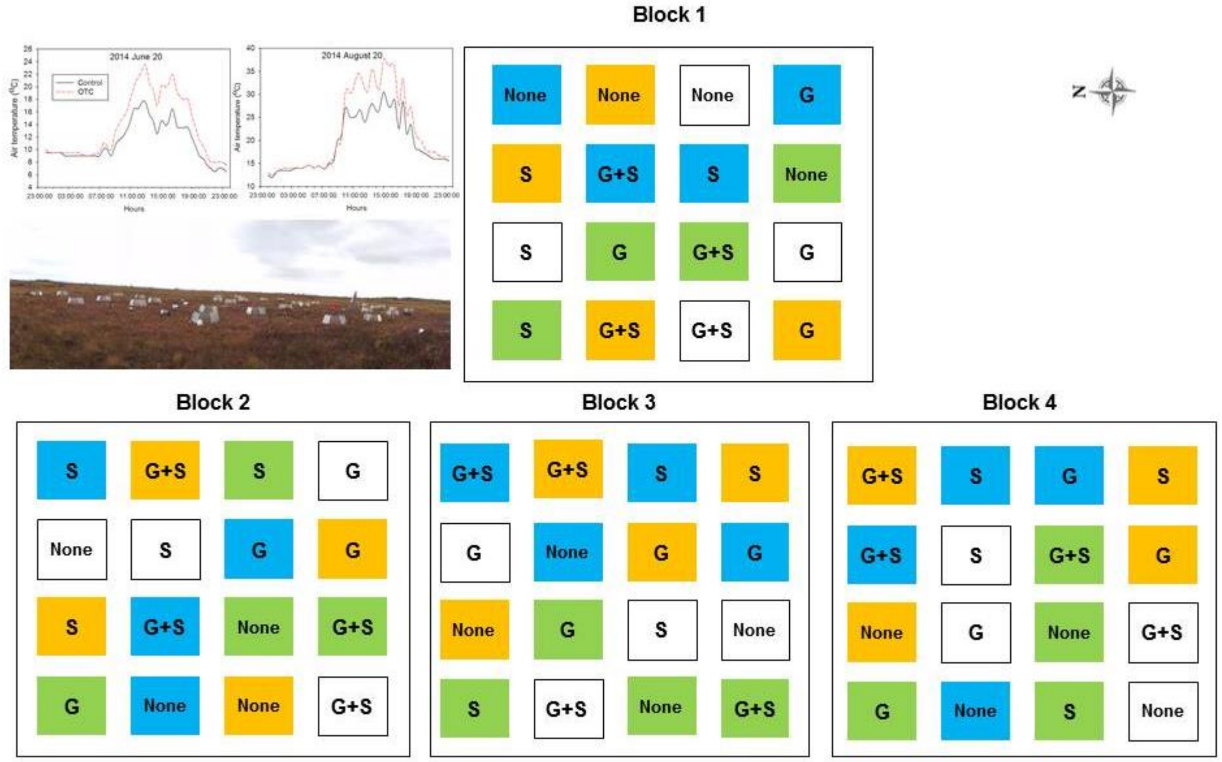

Supplementary Figure 2 Seasonal variations of ecosystem respiration, CH_4, and N_2O fluxes for control and nitrogen added plots (means ± SE), symbols and lines in red color: warmed by OTCs; symbols and lines in black color: ambient temperature. G+S, graminoids and shrubs both present; G, graminoids only present; S, shrubs only present; None, no vascular vegetation present.

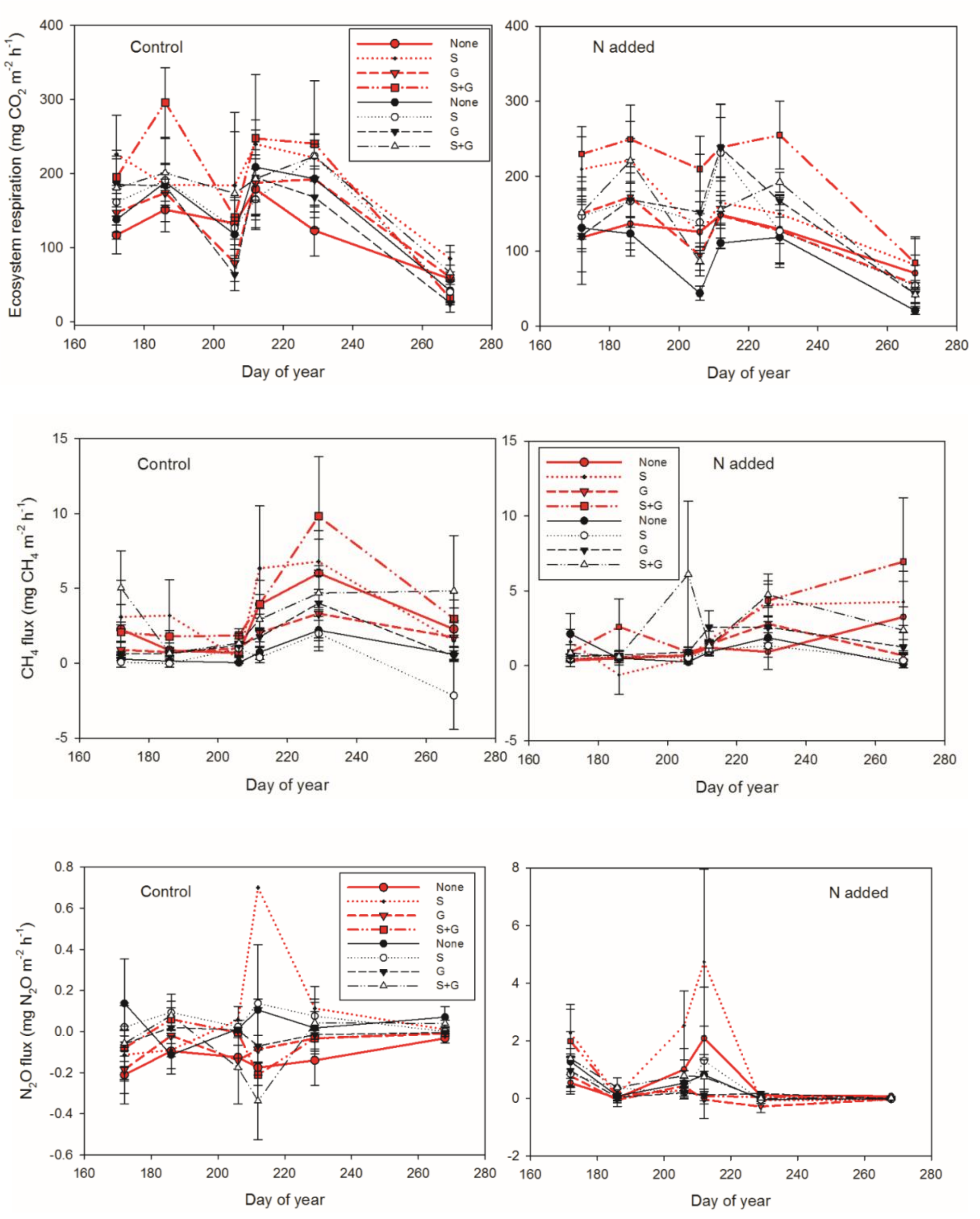

Supplementary Figure 3 Ecosystem respiration (a), methane (b), and nitrous oxide (c) fluxes observed during the manipulation experiment year of 2014. Data shown are means ± SE across all sampling dates. G+S, graminoids and shrubs both present; G, graminoids only present; S, shrubs only present; None, no vascular vegetation present. Note: for all plots, the substrate layer of *Sphagnum* mosses remained intact.

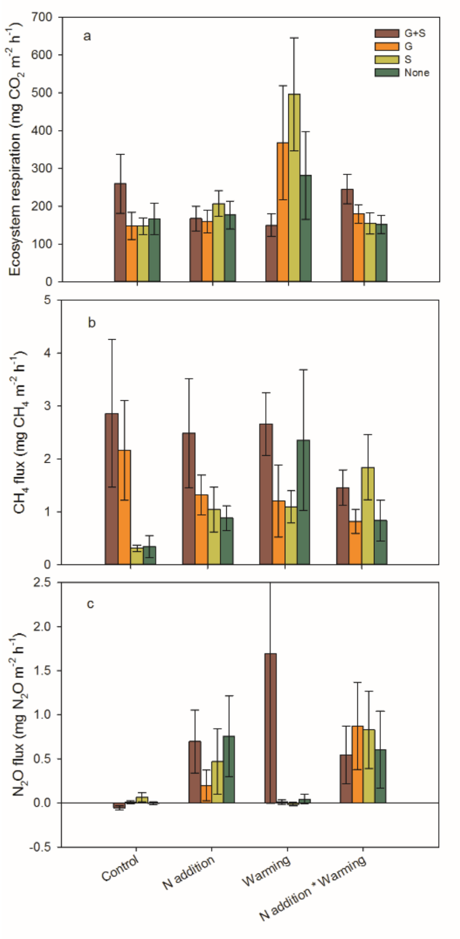

Supplementary Table 1 Soil temperature, recorded at depths of 5 cm (T_5) and 20 cm (T_{20}), and water table depth (WTD) at the study site. Graminoids+Shrubs, graminoids and shrubs both present; Graminoids, graminoids only present; Shrubs, shrubs only present; None, no vascular vegetation present.

Here we showed our manually measured soil temperature at 5 cm and 20 cm depth rather than air temperature, because soil temperature is thought to be the critical abiotic factors that influence GHG emissions at upper or lower layer of the mean water table depth (i.e., ~10 cm below the ground), representing the temperature for aerobic or anaerobic condition respectively.

Vegetation plus other manipulation	T_5		T_{20}		WTD	
	Mean	SE	Mean	SE	Mean	SE
Control						
Shrubs+Graminoids	18.1	0.7	15.5	0.5	-9.1	0.9
Shrubs	17.8	0.7	15.3	0.5	-9.3	0.8
Graminoids	18.7	0.9	15.7	0.5	-8. 8	0.8
None	17.5	0.8	15.2	0.5	-10.8	0.9
N addition						
Shrubs+Graminoids	18.1	1.0	15.6	0.5	-9.0	0.8
Shrubs	17.8	0.7	15. 5	0.5	-8.6	0.8
Graminoids	17.5	0.7	15.5	0.5	-10.0	0.8
None	17.7	0.7	15.5	0.5	-9.3	0.8
Warming						
Shrubs+Graminoids	19.9	0.8	16.2	0.5	-9.2	0.8
Shrubs	18.5	0.8	15.8	0.5	-11.4	1.5
Graminoids	18.7	0.8	15.9	0.5	-10.2	0.8
None	19.1	0.7	15.8	0.5	-9.5	0.8
N addition × Warming						
Shrubs+Graminoids	18.4	0.7	15.75	0.50	-9.51	0.97
Shrubs	19.8	0.8	15.96	0.50	-9.98	0.84
Graminoids	19.8	0.8	15.83	0.49	-10.12	0.73
None	18.7	0.8	15.79	0.51	-9.54	0.88

Supplementary Table 2 Statistical analysis of the effects of, and interactions between warming, N addition, and present/absence of plant functional groups, i.e., Shrubs and Graminoids, on soil temperature and water table depth. T_5, T_{20}, soil temperature at 5, 20 cm depths; WTD, water table depth. *df*: degree of freedom; *F*: F value; n: number of samples.

Manipulated variables	Statistical parameters for dependent variables								
	T_5			T_{20}			WTD		
	df	*F*	*P*	*df*	*F*	*P*	*df*	*F*	*P*
	n = 384			n = 384			n = 384		
N added/no N added	1	0.05	0.83		<0.001	0.98		0.15	0.70
Warmed/ambient temperature	1	**18.2**	**<0.001**		**29.8**	**<0.001**		0.66	0.42
Graminoids presence/absence	1	1.06	0.31		**5.23**	**0.03**		0.19	0.66
Shrubs presence/absence	1	0.07	0.79		0.87	0.36		0.15	0.70
N added × Warmed	1	0.43	0.52		0.38	0.54		<0.001	0.98
N added × Graminoids	1	1.57	0.22		2.72	0.11		0.80	0.38
N added × Shrubs	1	0.007	0.94		0.11	0.74		0.087	0.77
Warmed × Graminoids	1	0.16	0.69		1.58	0.22		0.002	0.96
Warmed × Shrubs	1	<0.001	0.99		0.15	0.70		0.42	0.52
Graminoids × Shrubs	1	0.31	0.58		0.29	0.59		0.21	0.65
N added × Warmed × Graminoids	1	0.001	0.97		0.20	0.66		0.11	0.74
N added × Warmed × Shrubs	1	0.93	0.34		1.21	0.28		0.009	0.92
Warmed × Graminoids × Shrubs	1	0.016	0.90		0.43	0.51		0.96	0.33
N added × Graminoids × Shrubs	1	1.69	0.20		0.26	0.61		0.003	0.96
N added × Warmed × Graminoids × Shrubs	1	**6.46**	**0.01**		1.42	0.24		0.52	0.47

Supplementary Table 3: Multivariate tests for the CO_2, CH_4, N_2O fluxes of repeated measures (6 times) ANOVA. Only results of Pillai's Trace were shown.

		CO_2		CH_4		N_2O	
	Hypothesis *df*	*F*	*Sig.*	*F*	*Sig.*	*F*	*Sig.*
time	5.000	13.380[b]	.001	7.062[b]	0.001	17.183[b]	0.002
time * N	5.000	1.113[b]	.424	.882[b]	0.503	9.057[b]	0.009
time * W	5.000	.720[b]	.627	1.679[b]	0.165	1.186[b]	0.414
time * Graminoids	5.000	.252[b]	.927	.834[b]	0.534	1.632[b]	0.283
time * Shrubs	5.000	.582[b]	.715	.151[b]	0.978	3.486[b]	0.08
time * N * W	5.000	.990[b]	.480	.534[b]	0.749	3.681[b]	0.072
time * N * Graminoids	5.000	.193[b]	.957	1.125[b]	0.365	2.005[b]	0.211
time * N * Shrubs	5.000	1.301[b]	.352	.550[b]	0.737	1.541[b]	0.305
time * W * Graminoids	5.000	.868[b]	.542	.666[b]	0.652	6.203[b]	0.023
time * W * Shrubs	5.000	1.005[b]	.472	.919[b]	0.48	9.059[b]	0.009
time * Graminoids * Shrubs	5.000	.455[b]	.799	2.226[b]	0.074	5.695[b]	0.028
time * N * W * Graminoids	5.000	.531[b]	.748	1.003[b]	0.431	--	--
time * N * W * Shrubs	5.000	.264[b]	.921	1.095[b]	0.38	--	--
time * N * Graminoids * Shrubs	5.000	1.042[b]	.455	.711[b]	0.619	--	--
time * W * Graminoids * Shrubs	5.000	.793[b]	.583	.468[b]	0.798	--	--

a. Design: Intercept + N + W + Graminoids + Shrubs + N * W + N * Graminoids + N * Shrubs + W * Graminoids + W * Shrubs + Graminoids* Shrubs + N * W * Graminoids + N * W * Shrubs + N * Graminoids * Shrubs + W * Graminoids * Shrubs + N * W * Graminoids * Shrubs

Within Subjects Design: time

b. Exact statistic

W: warming; N: nitrogen addition; Graminoids: graminoids present or not; Shrubs: shrubs present or not.
