# Supplementary material for: Soil nitrogen determines greenhouse gas emissions from northern peatlands under concurrent warming and vegetation shifting
Source: Commun Biol. 2019 Apr 18;2:132. doi: 10.1038/s42003-019-0370-1 (PMC6472372; doi:10.1038/s42003-019-0370-1)
Supplement: Supplementary file 2 — Description of Additional Supplementary Files [file 42003_2019_370_MOESM2_ESM.docx]

**Legend for Supplementary Data**

This is all the source data used in Figure 1, Figure 2 and Figure 3 in the main text.

The legend for the supplementary data is described as follows.

CH4 for methane (CH_4_)

CO2 for carbon dioxide (CO_2_)

N2O for nitrous oxide (N_2_O)

N addition means Nitrogen addition

DOC means Dissolved Organic Carbon

TN means Total Nitrogen

DOC/TN means the ratio between DOC and TN

Specific UV absorbance (SUVA254) means the specific UV absorbance at 254 nm

A250/a365 means the ratio between the UV absorbance at 250 nm and the UV absorbance at 365 nm

“Graminoids+Shrubs” represents graminoids and shrubs both present

“Graminoids” represents graminoids only present

“Shrubs” represents shrubs only present

None represents no vascular vegetation present.
