# Supplementary material for: Soil nitrogen determines greenhouse gas emissions from northern peatlands under concurrent warming and vegetation shifting
Source: Commun Biol. 2019 Apr 18;2:132. doi: 10.1038/s42003-019-0370-1 (PMC6472372; doi:10.1038/s42003-019-0370-1)
Supplement: Supplementary file 3 — Reporting Summary [file 42003_2019_370_MOESM3_ESM.pdf]

natureresearch

Corresponding author(s): Jianghua Wu

Last updated by author(s): Mar 4, 2019

Reporting Summary

Nature Research wishes to improve the reproducibility of the work that we publish. This form provides structure for consistency and transparency in reporting. For further information on Nature Research policies, see Authors & Referees and the Editorial Policy Checklist.

Statistics

For all statistical analyses, confirm that the following items are present in the figure legend, table legend, main text, or Methods section.

n/a	Confirmed	
☐	☒	The exact sample size (*n*) for each experimental group/condition, given as a discrete number and unit of measurement
☐	☒	A statement on whether measurements were taken from distinct samples or whether the same sample was measured repeatedly
☐	☒	The statistical test(s) used AND whether they are one- or two-sided<br>*Only common tests should be described solely by name; describe more complex techniques in the Methods section.*
☐	☒	A description of all covariates tested
☐	☒	A description of any assumptions or corrections, such as tests of normality and adjustment for multiple comparisons
☐	☒	A full description of the statistical parameters including central tendency (e.g. means) or other basic estimates (e.g. regression coefficient) AND variation (e.g. standard deviation) or associated estimates of uncertainty (e.g. confidence intervals)
☐	☒	For null hypothesis testing, the test statistic (e.g. F, t, r) with confidence intervals, effect sizes, degrees of freedom and P value noted<br>*Give P values as exact values whenever suitable.*
☒	☐	For Bayesian analysis, information on the choice of priors and Markov chain Monte Carlo settings
☒	☐	For hierarchical and complex designs, identification of the appropriate level for tests and full reporting of outcomes
☒	☐	Estimates of effect sizes (e.g. Cohen's d, Pearson's r), indicating how they were calculated

Our web collection on statistics for biologists contains articles on many of the points above.

Software and code

Policy information about availability of computer code

Data collection	N/A
Data analysis	N/A

For manuscripts utilizing custom algorithms or software that are central to the research but not yet described in published literature, software must be made available to editors/reviewers. We strongly encourage code deposition in a community repository (e.g. GitHub). See the Nature Research guidelines for submitting code & software for further information.

Data

Policy information about availability of data

All manuscripts must include a data availability statement. This statement should provide the following information, where applicable:

- Accession codes, unique identifiers, or web links for publicly available datasets
- A list of figures that have associated raw data
- A description of any restrictions on data availability

The data that support the findings of this study are available from the corresponding author upon reasonable request.

Field-specific reporting

Please select the one below that is the best fit for your research. If you are not sure, read the appropriate sections before making your selection.

☐ Life sciences ☐ Behavioural & social sciences ☒ Ecological, evolutionary & environmental sciences

For a reference copy of the document with all sections, see nature.com/documents/nr-reporting-summary-flat.pdf

Ecological, evolutionary & environmental sciences study design

All studies must disclose on these points even when the disclosure is negative.

Study description	We manipulated the temperature, plant community composition, and nitrogen (N) content of boreal peatland plots to determine how these factors affect greenhouse gas emissions. A factorial design comprising manipulation of temperature (warmed vs. ambient temperature), nitrogen (N; N addition vs. no N addition), and plant community composition (removal of graminoids only vs. removal of shrubs only vs. removal of both graminoids and shrubs vs. no vegetation removed) was established in the spring of 2014. The layout was 2 (Warming and ambient temperature) × 2 (N addition and no N addition) × 2 (shrubs present or absent) × 2 (graminoids present or absent) = 16 treatments. The experimental site comprised four blocks with 6 m apart from each other, and each block contained one each of all 16 treatments, randomly distributed (n = 64)
Research sample	For ecosystem respiration (Reco), CH4 and N2O fluxes, gas samples were collected using opaque chambers 50 cm in height and 26.3 cm in diameter, fitted to the groove of the PVC collar, covered with aluminum foil to reduce any solar heating effect, and equipped with a capillary tube to maintain atmospheric pressure inside the chamber when sampling. Samples were taken immediately upon closure of the chambers and at 10 min., 20 min. and 30 min. after closure. Flux was calculated by linear regression using all four measurements sampled during the 30 minutes 39,57. Because the light was blocked by the opaque chamber, no photosynthesis occurred. Thus, the flux calculated based on the change of CO2 concentration inside the opaque chamber can be considered to be the ecosystem respiration. Gas samples (25 mL) were taken from the chamber headspace using a gas syringe and injected into pre-evacuated 12 mL Exetainer vials (Labco, Lampeter, UK) for storage prior to analysis. Concentrations of CO2, CH4 and N2O were analyzed by gas chromatography using a Scion 456-GC (gas chromatograph; Bruker, Milton, Canada) equipped with a thermal conductivity detector for CO2, a flame ionization detector for CH4, and an electron capture detector for N2O. For each sample, 5 mL of gas was injected into the chromatograph using an Autosampler (Combi PAL, Milton, Canada). The gas concentration was calculated using a calibration curve based on two certified standard gases, comprising 378 ppm and 0.303% CO2 (i.e. 3030 ppm), 2.52 and 17.7 ppm CH4, and 0.770 and 7.63 ppm N2O (Air Liquide, Canada). All fluxes were adjusted for field sampling temperature, headspace volume and chamber area 62.
Sampling strategy	In each sampling plot, a PVC (polyvinyl chloride) collar with an inner diameter of 26 cm was permanently inserted into the peat to a depth of 10 cm in the spring of 2014. The upper part of the collar features a groove to accommodate the water seal needed for the chamber measurements. We had in total six sampling campaigns at approximately three-week intervals from June to October 2015. During each sampling campaign, measurements were done for all the plots and conducted between 10:00-15:00 local time during 2 to 4 days to avoid rainfall.
Data collection	For ecosystem respiration (Reco), CH4 and N2O fluxes, gas samples were collected using opaque chambers 50 cm in height and 26.3 cm in diameter, fitted to the groove of the PVC collar, covered with aluminum foil to reduce any solar heating effect, and equipped with a capillary tube to maintain atmospheric pressure inside the chamber when sampling. Samples were taken immediately upon closure of the chambers and at 10 min, 20 min and 30 min after closure. Two undergraduate summer RAs under the supervision of Dr. Jianghua Wu collected the samples and analyzed the samples in the lab during the summer of 2015.
Timing and spatial scale	Measurements were conducted between 10:00-15:00 local time, at approximately three week intervals from June to October 2015.
Data exclusions	N/A
Reproducibility	We continue to run the experiments to see how long-term treatments would affect greenhouse gas emissions in boreal peatlands.
Randomization	The treatments were randomly distributed in the field and four replicates for each treatment were employed.
Blinding	N/A

Did the study involve field work? ☒ Yes ☐ No

Field work, collection and transport

Field conditions	The climate is oceanic temperate, with an annual rainfall of 1340 mm and annual average temperature of 5 °C (1981–2010) 57. The mean pH (1:5 soil/water) at the site was 4.5 ± 0.01, and the mean peat depth of 3 m was derived from 3 random peat depth measurements at the site before the experiment was established. The site represents the typical type of peatland found on the island of Newfoundland, where the vegetation consists of an approximately equal biomass of graminoids (Trichophorum cespitosum, Carex chordorrhiza) and dwarf shrubs (Gaylussacia baccata, Rhododendron groenlandicum, Andromeda glaucophylla, Ledum palustre ssp.), with Sphagnum mosses (Sphagnum spp., Hylocomium splendens, Aulacomnium turgidum) providing the main matrix 39,57.
Location	Our research site is located in an area of oligogenic, ombrotrophic blanket bog, in Robinsons, western Newfoundland, Canada (48°15′46″N, 58°39′ 21″W).
Access and import/export	We accessed the sampling plots through the installed boardwalks. We walked to the sampling site.
Disturbance	Care was taken during collar insertion to minimize disturbance and to avoid severance of large plant roots. Boardwalks were installed to prevent any damage to the vegetation and disturbance to peat gas storage or, emissions during site visits.

Reporting for specific materials, systems and methods

We require information from authors about some types of materials, experimental systems and methods used in many studies. Here, indicate whether each material, system or method listed is relevant to your study. If you are not sure if a list item applies to your research, read the appropriate section before selecting a response.

Materials & experimental systems

n/a	Involved in the study
☒	☐ Antibodies
☒	☐ Eukaryotic cell lines
☒	☐ Palaeontology
☒	☐ Animals and other organisms
☒	☐ Human research participants
☒	☐ Clinical data

Methods

n/a	Involved in the study
☒	☐ ChIP-seq
☒	☐ Flow cytometry
☒	☐ MRI-based neuroimaging
